# Effectiveness of Two Web-Based Interventions for Chronic Cancer-Related Fatigue Compared to an Active Control Condition: Results of the “Fitter na kanker” Randomized Controlled Trial

**DOI:** 10.2196/jmir.7180

**Published:** 2017-10-19

**Authors:** Fieke Z Bruggeman-Everts, Marije D J Wolvers, Rens van de Schoot, Miriam M R Vollenbroek-Hutten, Marije L Van der Lee

**Affiliations:** ^1^ Helen Dowling Instituut Scientific Research Department Bilthoven Netherlands; ^2^ Telemedicine Group Faculty of Electrical Engineering, Mathematics, and Computer Science University of Twente Enschede Netherlands; ^3^ Roessingh Research and Development Telemedicine Group Enschede Netherlands; ^4^ Telemedicine Group Faculty of Electrical Engineering Mathematics and Computer Science University of Twente Enschede Netherlands; ^5^ Department of Methods and Statistics Utrecht University Utrecht Netherlands; ^6^ North-West University Vanderbijlpark South Africa

**Keywords:** fatigue, cancer survivors, Internet interventions, mindfulness-based cognitive therapy, physiotherapy, accelerometry, latent growth analysis, implementation, RCT

## Abstract

**Background:**

Approximately one third of all patients who have been successfully treated for cancer suffer from chronic cancer-related fatigue (CCRF). Effective and easily accessible interventions are needed for these patients.

**Objective:**

The current paper reports on the results of a 3-armed randomized controlled trial investigating the clinical effectiveness of two different guided Web-based interventions for reducing CCRF compared to an active control condition.

**Methods:**

Severely fatigued cancer survivors were recruited via online and offline channels, and self-registered on an open-access website. After eligibility checks, 167 participants were randomized via an embedded automated randomization function into: (1) physiotherapist-guided Ambulant Activity Feedback (AAF) therapy encompassing the use of an accelerometer (n=62); (2) psychologist-guided Web-based mindfulness-based cognitive therapy (eMBCT; n=55); or (3) an unguided active control condition receiving psycho-educational emails (n=50). All interventions lasted nine weeks. Fatigue severity was self-assessed using the Checklist Individual Strength - Fatigue Severity subscale (primary outcome) six times from baseline (T0b) to six months (T2). Mental health was self-assessed three times using the Hospital Anxiety and Depression Scale and Positive and Negative Affect Schedule (secondary outcome). Treatment dropout was investigated.

**Results:**

Multiple group latent growth curve analysis, corrected for individual time between assessments, showed that fatigue severity decreased significantly more in the AAF and eMBCT groups compared to the psycho-educational group. The analyses were checked by a researcher who was blind to allocation. Clinically relevant changes in fatigue severity were observed in 66% (41/62) of patients in AAF, 49% (27/55) of patients in eMBCT, and 12% (6/50) of patients in psycho-education. Dropout was 18% (11/62) in AAF, mainly due to technical problems and poor usability of the accelerometer, and 38% (21/55) in eMBCT, mainly due to the perceived high intensity of the program.

**Conclusions:**

Both the AAF and eMBCT interventions are effective for managing fatigue severity compared to receiving psycho-educational emails.

**Trial Registration:**

Trialregister.nl NTR3483; http://www.trialregister.nl/trialreg/admin/rctview.asp?TC=3483 (Archived by WebCite at http://www.webcitation.org/6NWZqon3o)

## Introduction

Cancer-related fatigue (CRF) is, “a distressing, persistent, subjective sense of physical, emotional, and/or cognitive tiredness or exhaustion related to cancer or cancer treatment that is not proportional to recent activity and interferes with usual functioning” [1]. In approximately 30% of the patients who have been successfully treated for cancer, severe fatigue persists for months or even years [2]. This persistent fatigue, termed chronic CRF (CCRF) is often accompanied by distress and poor mental health [1,3].

Physical activity interventions and psychosocial interventions specifically designed to reduce CCRF have been shown to be effective [4-9]. Readily accessible interventions are needed for patients who do not have the energy or time to travel to a specialized health care institute [10,11], and so we have developed two different Web-based interventions aimed at reducing CCRF: (1) a physiotherapist-guided Ambulant Activity Feedback (AAF) [12], and (2) a psychologist-guided Web-based Mindfulness-Based Cognitive Therapy (eMBCT) [13]. Wolvers et al [14] detail an elaboration on the theoretical models underlying these interventions.

The overall aim of the project *More fit after cancer* (in Dutch *Fitter na kanker*, hereafter referred to as the FNK trial) was to study the effectiveness, effect predictors, and mediators of AAF and eMBCT in comparison to a minimal active control condition that consisted of emails with psycho-education about CCRF [14]. This paper reports on the clinical effectiveness of AAF and eMBCT in reducing fatigue severity and improving mental health in severely fatigued cancer survivors, compared to psycho-education. We hypothesized that fatigue severity would be reduced more, and mental health would be increased more in AAF and eMBCT compared to PE, between baseline and six-month follow-up.

## Methods

### Patients and Setting

In our previous article [14] we provided a detailed description of the methods of this trial. Severely fatigued cancer survivors were recruited via online and offline channels (via patient organizations, walk-in consultation services, social media, newspapers, and health care professionals; see Multimedia Appendix 1), inviting them to follow a Web-based intervention in a research setting for their fatigue, and invited them to register on an open-access website [15,16]. To recruit a group of participants with open expectations, we did not specify the exact content of the interventions in the advertisements. See Multimedia Appendix 2 (advertisement) and Multimedia Appendix 3 (informed consent) for the information given during recruitment.

Participants (all cancer types included) had finished curative-intent cancer treatment (with the exception of hormonal treatment, as this is often low intensity and may last up to five years) at least three months previously, and had been suffering from severe fatigue ever since (≥35 on the Checklist Individual Strength - Fatigue Severity [CIS-FS] subscale) [7,17]. Participants had no current or former severe psychiatric morbidity (eg, suicidal ideation, psychosis, or schizophrenia), were >19 years old, and were at least 18 years old at disease onset. For external validity purpose, nontreatable comorbid somatic diseases that were possible causes for fatigue (eg, rheumatoid arthritis, diabetes, myocardial damage) were not excluded, and were registered during the study. We chose not to statistically control for these comorbidities, but to check whether comorbidities were equally divided between the conditions (see Multimedia Appendix 4). We contacted each participant’s medical doctor (general practitioner, oncologist, or other medical specialist) after participant consent was obtained, to check for psychiatric morbidity and whether curative intent cancer treatment had finished at least three months previously.

**Figure 1:**
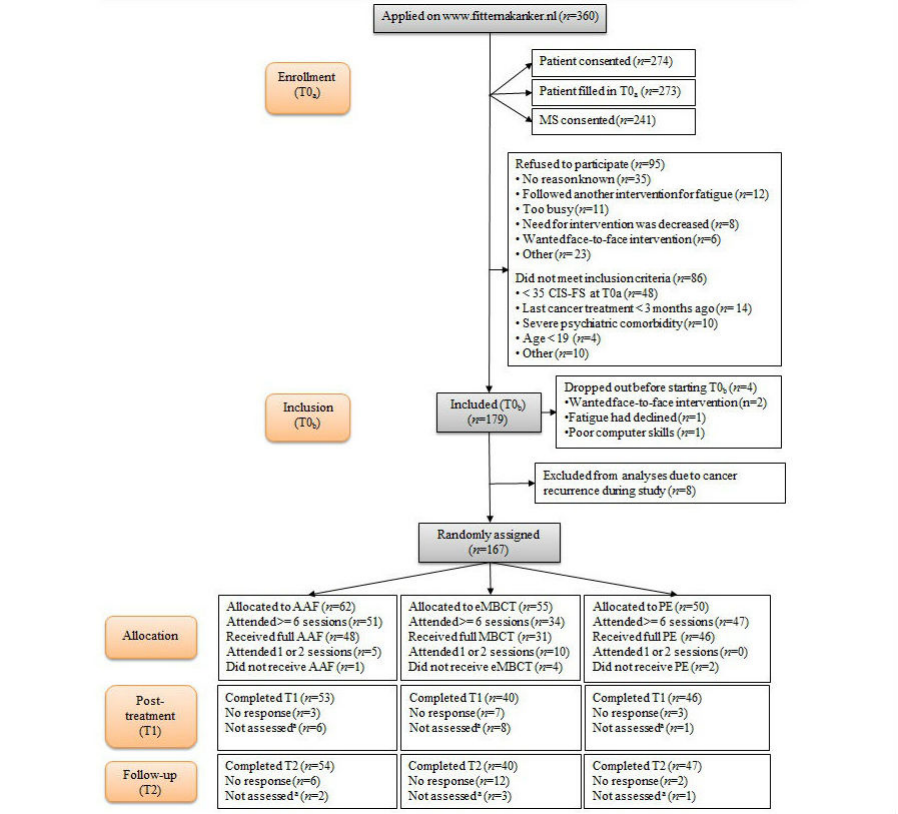
Flowchart of Fitter na kanker trial. The last five participants were not included in the analysis, as they were still in the trial at time of analysis. AAF: Ambulant Activity Feedback; CIS-FS: Checklist Individual Strength - Fatigue Severity subscale; eMBCT: Web-based Mindfulness-Based Cognitive Therapy; MS: medical specialist; PE: psycho-education.

We aimed to include 330 participants to be able to study working mechanisms, in addition to the effectiveness of the interventions. Despite persistent recruitment efforts and an extension of the recruitment period by three months, this number proved infeasible as we had to exclude more patients than anticipated (see Figure 1). However, we continued recruiting until we had enough participants to study the effectiveness with enough power; namely 55 participants per condition [14].

### Trial Design

Participants were randomized to one of three conditions by a computerized tool [14], which included two experimental conditions: (1) AAF and (2) eMBCT; or (3) an active control condition in which participants received psycho-education. The intervention period was nine weeks for all three conditions. The primary outcome was self-perceived fatigue severity measured after the eligibility check (T0_b_; baseline), three times during the intervention (M3, M6, M9), two weeks after completion of the interventions (T1), and six months after baseline (T2; primary outcome). The secondary outcome was mental health, measured at recruitment (T0_a_), T1, and T2. All outcomes were self-reported and Web-assessed. Participants were reminded to complete the measurements twice, and at T2 participants were also reminded by telephone. Dropouts from the treatment groups were interviewed by telephone to inquire about their reasons for dropping out.

### Randomization, Masking, and Blinding

We have described the randomization process in detail in our trial article [14]. Randomization was carried out blind via a script embedded in the researchers’ Web portal and used the random function of *php [rand(1,3)]* [18]. The researchers could neither influence nor predict the outcome of the randomization process. Due to an error in the website’s randomization algorithm, allocation was temporarily dependent on the number of participants who were allocated at the same time between January 14, 2014 and July 15, 2014 (see Wolvers et al [14] for more information). This issue resulted in unequal sample sizes for the conditions. We argue that the participants were randomly assigned, as it was not the researchers’ decision regarding how many participants were allocated at the same time. Neither researchers, participants, nor therapists were blind to treatment, as the medical ethical committee insisted that we announced the minimal intervention as our control group. An independent statistician (RvdS) was blind to allocation while checking all analyses. We did not specify the exact content of the interventions in the advertisements, in an effort to limit influencing expectations before the trial began.

### Interventions

See Multimedia Appendix 5 for additional screen shots of all interventions studied. The eMBCT is a Web-based psychologist-guided intervention, which follows the MBCT protocol specifically designed for CCRF [19,20]. eMBCT aims to change the patient’s behavioral and cognitive reactions to cancer-related stressors, including fatigue itself [5,19,21]. Following the original eMBCT protocol, participants who were randomized into eMBCT were diagnosed according to the Diagnostic and Statistical Manual of Mental Disorders, Fourth Edition, Text Revision (DSM-IV-TR) [22]. The intervention’s time-investment involves reading the weekly information, doing mindfulness exercises while listening to the MP3 files, filling out logs with their experiences, reading the weekly feedback of the therapist, and replying to this feedback by email weekly. The time investment for participating the eMBCT was estimated to be four hours per week (on average) for nine weeks. Participants could not continue with a following session before they had registered their experience with a homework assignment from the previous week. Bruggeman-Everts et al [13] have published a pilot study on the effectiveness of eMBCT and a detailed description of the eMBCT protocol, setting, and development.

The AAF consists of a home-based physiotherapist-guided protocol in which participants use an accelerometer to gain insight into their physical activity patterns, and increase or balance their daily activities in ways that improve their energy levels [5,23]. The time investment for the AAF intervention is estimated to be three hours per week (on average) for nine weeks. The time-investment involves taking notice of the Personal Digital Assistant messages, responding to these messages by changing physical activity, reading the weekly feedback from the physiotherapist, reporting experiences, and replying to the feedback by email. Participants could not continue with a following session before they had registered their experience with a homework assignment from the previous week. See Wolvers and Vollenbroek-Hutten [12] for a detailed description of the development of AAF.

Patients in the psycho-education condition received psycho-educational emails describing possible causes of fatigue, sleep hygiene, balancing energy during the day, and how to cope with worrying thoughts. We estimated that patients dedicated ten minutes per week to the nine-week minimal control intervention. The intervention involves reading the psycho-education information in no-reply emails. Whether participants had indeed read the psycho-education information was not checked, as asking participants was considered unreliable. This psycho-education information was derived from the eMBCT protocol for CCRF [13,19], and was included in the current eMBCT and AAF protocols, so participants in all three conditions were given the same PE.

### Outcomes

The primary outcome of fatigue severity was measured using the CIS-FS [7,17], which consists of eight items that are rated on a seven-point Likert scale (range 8-56, Cronbach alpha=0.84). The CIS closely resembles the Multidimensional Fatigue Inventory [24,25]. The secondary outcome was the concept of *mental health* measured using both negatively and positively framed questionnaires [26]: the Positive and Negative Affect Schedule [27,28] was used to measure Positive Affect (PA; range 10-50, Cronbach alpha *=* 0.90) and Negative Affect (NA; range 10-50, Cronbach alpha *=* 0.89); and the Hospital Anxiety and Depression Scale (HADS; range 0-42, Cronbach alpha=0.88) [29-31] was used to measure distress.

Baseline characteristics were assessed, including demographics, medical history, and help received in the past. Participants could only continue with the next week’s exercises after finishing the previous, so adherence was calculated based on the week number that participants had reached. The proportion of nonadherence was based on the number of participants who dropped out of the intervention before completing 6 weeks of the protocol (ie, intended usage) [14].

### Data Analyses

First, analysis of variance (ANOVA) and Chi-square tests were performed to: (1) check for differences in baseline characteristics between all conditions; and (2) check whether baseline variables correlated with missing data patterns, to check if data was randomly missing. The significance level was set at *P*<.01 to correct for multiple testing; this resulted in no auxiliary variables or covariates being included in the model. Outcome measures were checked for normality and outliers, and resulted in no modifications being made. These analyses were performed in SPSS Version 23 for Windows (SPSS Inc, Chicago, IL).

Second, Longitudinal Growth Modeling (LGM) was performed to test which model best fit the longitudinal data of the outcome measures (CIS-FS, HADS, PA, and NA) using Mplus version 7.31 [32]: (1) a linear versus linear and quadratic slope; (2) one slope versus a piece-wise model with two slopes (piece-wise only for CIS-FS); and (3) with versus without individual time scores (the exact time points when a participant filled in the assessment). See Multimedia Appendix 6 for the procedure of selecting the best fitting model for CIS-FS. Next, we studied the effectiveness of AAF and eMBCT compared to PE by testing whether the trajectories of the best fitting model significantly differed between the three conditions by applying Wald testing (for linear slopes) or Chi-square difference testing (for linear and quadratic slopes). This was done on an intention-to-treat basis (thus including adherent and nonadherent participants) and we checked whether the results for CIS-FS changed when only including participants who were adherent to treatment.

Third, to measure the clinical importance in addition to statistical significance, the proportion of participants who were clinically relevantly changed on CIS-FS was calculated for each condition, using the reliable change index (RCI) [33,34]. See Multimedia Appendix 7 for the calculations of the proportion of clinically relevantly changed participants. We used a clinical cut-off score of a normative group (CIS-FS< 28.0 [35]) which consisted of nonfatigued breast cancer survivors [35]. In our trial design paper [14], we suggested the use of a normative group of women without a history of breast cancer [35], however we think it is better to use a normative group that indeed had a history of cancer, as it is such a disruptive illness, and comparing the group to healthy subjects would be less informative. The proportions of participants who had *recovered* (passed both the cut-off score of the normative group and the RCI criteria), *improved* (passed the RCI criteria in the direction of fatigue reduction), were *unchanged* (did not pass the RCI criteria), or *deteriorated* (passed the RCI in the direction of fatigue increase) were all calculated.

Finally, notes and quotations from the telephone interviews with nonadherent participants were analyzed by close reading, followed by clustering of emerging themes concerning reasons for dropping out. ANOVA and Chi-square tests were performed to identify differences between adherent and nonadherent participants. The proportion of nonadherent participants was calculated.

### Ethical Approval

All participants gave written informed consent prior to their inclusion in the study. This trial was approved by the Twente Medical Ethical Committee (Enschede, The Netherlands), number P12-26, and was registered in The Netherlands National Trial Register under number NTR3483 [36].

## Results

### Patients

Between March 2013 and June 2015, 360 people applied on the website to participate (see Figure 1 for flowchart). See Multimedia Appendix 1 for details about recruitment over the course of time. Applicants for the FNK-trial had heard about the project via family or friends (16.1%, 58/360), via patient societies (12.5%, 45/360), through a search on the Internet (11.7%, 42/360), via health professionals (5.8%, 21/360), or otherwise (unknown; 53.8%, 194/360).

We excluded 23.8% (86/360) of the applicants (mean age=56.3 years, standard deviation [SD]=13.3; 59%, 51/86 women) for the reasons given in Figure 1, and another 26.4% (95/360) declined to participate (mean age=58.0 years, SD=12.7; 67%, 64/95 women) before the eligibility criteria were checked. Eventually, 179 participants were included (see Multimedia Appendix 4 for baseline characteristics); of these, four participants dropped out before filling in T0_b_(mean age=60.5 years, SD=7.7; 75%, 3/4 women), and eight participants were excluded from analyses due to cancer recurrence during the study (mean age=59.8 years, SD=6.5; 50%, 4/8 women), leaving 167 participants for analyses.

Participants were randomized to one of the three conditions: (1) AAF (n=62), (2) eMBCT (n=55), or (3) psycho-education (n=50). All participants in the eMBCT group met the DSM-IV-TR criteria for undifferentiated somatoform disorder, of whom 4 of 55 (7%) were additionally diagnosed with a sleeping disorder, 7 (13%) experienced work-related psychosocial problems, and 6 (11%) suffered from problems in their peer-support group.

### Effectiveness

**Figure 2:**
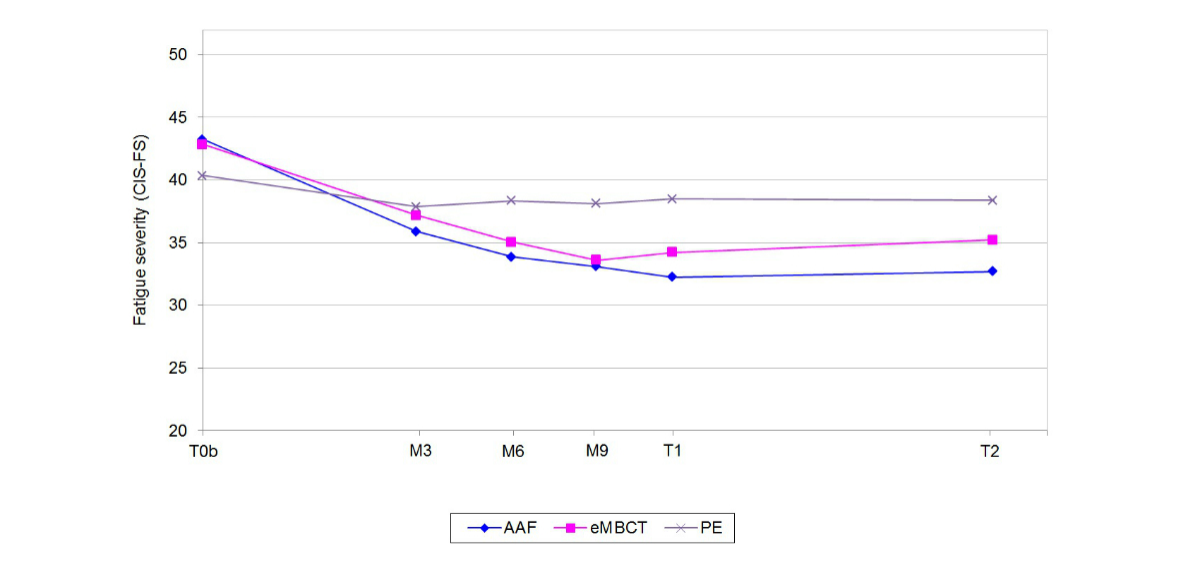
Sample means of fatigue severity (CIS-FS) for all three conditions (n=167). On the x-axis, the mean of timescores between T0b and M3, M6, M9, T1 and T2 are shown. Please note that the model included individual time scores. The average timescores (denoted in weeks, with standard deviations between brackets) between T0b and M3, M6, M9, T1, and T2 were 7.6 (2.4), 11.0 (2.8), 14.0 (2.6), 16.7 (3.2), and 28.1 (1.9), respectively. See Multimedia Appendix 6 for the average distribution of individual timescores between T0b and T2.

Model selection for CIS-FS showed that a model with both linear and quadratic slopes, individual times cores, freely estimated mean and slope variances, and residual variances fixed to be equal between conditions best fit the data. Figure 2 shows the sample means of CIS-FS between T0_b_ and T2 per condition.

Chi-square difference testing (see Table 1), with linear and quadratic slopes fixed to be equal between conditions, showed that the CIS-FS trajectories differed between all three conditions (*χ*^2^(4)=27.63, *P*<.001). More specifically, the trajectories of AAF and psycho-education differed (*χ*^2^(2)=28.28, *P*<.001), and eMBCT and psycho-education differed (*χ*^2^(2)=10.89, *P*=.004), while the trajectories of AAF and eMBCT were equal (*χ*^2^(2)=2.19, *P*=.34). When only including adherent participants (n=132), the results were similar: the slopes of AAF and eMBCT were equal (*χ*^2^(2)=0.991, *P*=.61), while the slopes of psycho-education and AAF differed (*χ*^2^(2)=28.109, *P*<.001), and psycho-education and eMBCT differed (*χ*^2^(2)=9.735, *P*=.008). The slope estimates indicated that CIS-FS decreased significantly more in the AAF and eMBCT conditions compared to the psycho-education condition.

The model fits for HADS, PA, and NA were best for linear models with individual time scores and slope variances fixed at zero. As shown in Table 2, the slopes in all three conditions were significantly different from zero: HADS and NA decreased, and PA increased. Table 3 presents the results of Wald testing, and shows that there were no significant differences in slopes between the HADS, PA, and NA between conditions.

### Clinically Relevant Change

**Figure 3:**
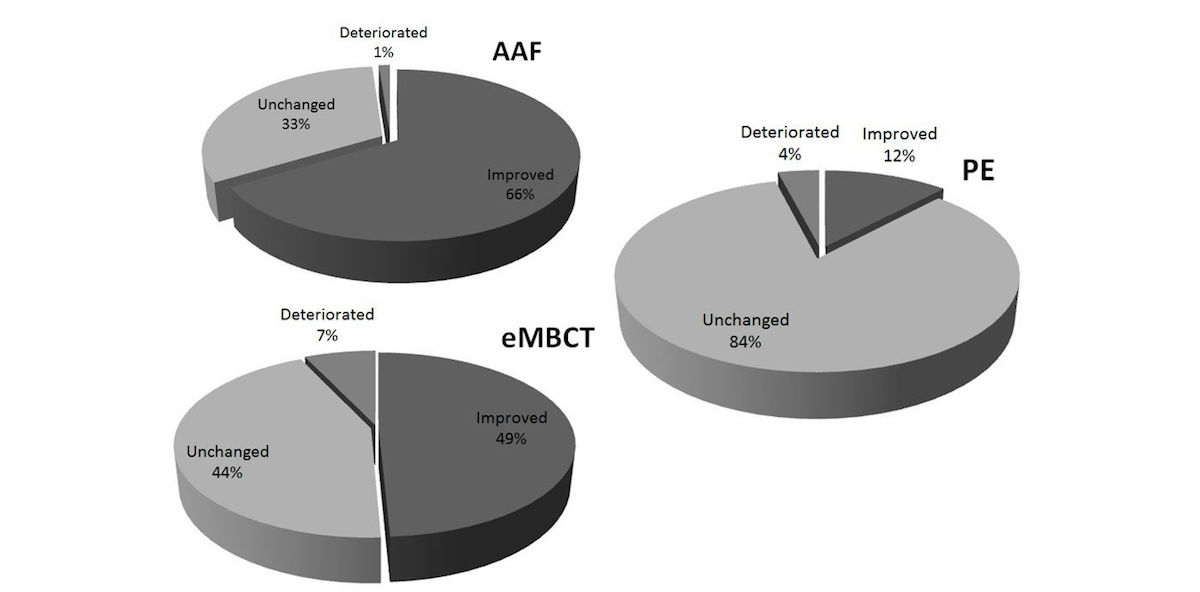
Proportions of clinically relevant changes (improved, unchanged, deteriorated) for each condition (intention-to-treat).

The proportion of *recovered* participants for AAF was 21% (13/62), for eMBCT was 9% (5/55), and for psycho-education was 2% (1/50). Of the adherent participants, 26% (13/51) recovered in the AAF condition, 6% (2/34) recovered in the eMBCT condition, and 2% (1/47) recovered in the psycho-education condition. Figure 3 shows the proportion of *improved, unchanged*, and *deteriorated* participants per condition. In the AAF condition 66% (41/62) improved, in the eMBCT condition 49% (27/55) improved, and in the psycho-education condition 12% (6/50) improved.

### Treatment Dropout

Nonadherence, the proportion of participants who dropped out the intervention before completing 6 weeks of the protocol, was 18% (11/62) in the AAF condition, 38% (21/55) in the eMBCT condition, and 6% (3/50) in the psycho-education condition. No differences in baseline characteristics were found between adherent and nonadherent participants.

Reasons for dropping out of AAF were mainly technical problems and poor usability of the accelerometer. Nonadherence of eMBCT was mainly due to the high intensity of the program, the exercises were considered too woolly, poor usability of the eMBCT portal, and difficulty in communicating in writing with the therapist. In both interventions, nonadherent participants said they stopped using the intervention due to a lack of confidence that the intervention would help them reduce fatigue. Other reasons were that fatigue had reduced considerably and treatment was no longer desired, or that participants preferred face-to-face contact instead.

**Table 1:** Results of the Chi-square testing of fatigue severity change (CIS-FS) between groups.

Hypothesis test	Results of Chi-square test
AAF = eMBCT = psycho-education	*χ*^2^(4)=27.63, *P*<.001	
AAF = psycho-education	*χ*^2^(2)=28.28, *P*<.001
eMBCT = psycho-education	*χ*^2^(2)=10.89, *P*=.004
AAF = eMBCT	*χ*^2^(2)=2.19, *P*=.34

**Table 2:** Model results of all outcome measurements. The mean intercepts and mean slope factors of all outcome measures with standard errors (in brackets) are presented.

Outcome	Condition	Intercept at T0_b_(I)	Linear slope factor (S)	Two-tailed *P* value of linear slope (*P*)	Quadratic slope factor (Q)	Two-tailed *P-* value of quadratic slope (*P*)
CIS-FS	AAF	42.838 (0.873)	-1.072 (0.162)	<.001	0.026 (0.005)	*P*<.001
	eMBCT	42.752 (1.020)	-0.876 (0.178)	<.001	0.022 (0.006)	*P*<.001
	Psycho-education	39.893 (1.243)	-0.208 (0.170)	.22	0.006 (0.006)	*P*=.31
HADS	AAF	13.237 (0.921)	-0.076 (0.017)	<.001	*N/A*	*N/A*
	eMBCT	13.903 (0.771)	-0.110 (0.022)	<.001	*N/A*	*N/A*
	Psycho-education	14.579 (1.012)	-0.083 (0.024)	<.001	*N/A*	*N/A*
PA	AAF	31.762 (0.939)	0.101 (0.022)	<.001	*N/A*	*N/A*
	eMBCT	28.995 (0.932)	0.156 (0.026)	<.001	*N/A*	*N/A*
	Psycho-education	29.422 (1.091)	0.128 (0.027)	<.001	*N/A*	*N/A*
NA	AAF	20.330 (0.931)	-0.068 (0.023)	.003	*N/A*	*N/A*
	eMBCT	20.718 (0.914)	-0.071 (0.032)	.03	*N/A*	*N/A*
	Psycho-education	20.805 (1.215)	-0.082 (0.029)	.004	*N/A*	*N/A*

**Table 3:** Results of Wald testing for differences between conditions (HADS, PA, and NA). All Wald tests were nonsignificant, indicating that there was no significant difference between the slopes of the conditions.

	Wald test	Result
HADS	AAF = psycho-education	0.067(1), *P*=.80
	eMBCT = psycho-education	0.665(1), *P*=.41
	AAF = eMBCT	1.491(1), *P*=.22
PA	AAF = psycho-education	0.599(1), *P*=.44
	eMBCT = psycho-education	0.573(1), *P*=.45
	AAF = eMBCT	2.640(1), *P*=.10
NA	AAF = psycho-education	0.148(1), *P*=.70
	eMBCT = psycho-education	0.065(1), *P*=.80
	AAF = eMBCT	0.006(1), *P*=.94

## Discussion

### Main Results

This is the first study to report on effectiveness of two guided Web-based interventions for CCRF. Using latent growth curve modeling, we found that AAF and eMBCT were significantly more effective in reducing fatigue severity than psycho-education. The proportions of participants that showed clinically relevant improvement were 66% (41/62) in the AAF condition, 49% (27/55) in the eMBCT condition, and 12% (6/50) in the psycho-education condition. Mental health improved in all three conditions. Treatment dropout was 18% (11/62) in the AAF condition and 38% (21/55) in the eMBCT condition. Reasons for dropping out of AAF were technical problems with the accelerometer, and eMBCT was considered to be too intensive. The AAF dropout rate is comparable to other online interventions [37], and in a previous pilot study in clinical practice we also found a dropout rate of 38.1% in eMBCT [13]. Taking these dropout rates into account, we can conclude that both AAF and eMBCT are effective interventions for reducing fatigue severity.

### Strengths and Limitations

Our study design has several strengths. First, in contrast to multivariate ANOVA, LGM allows the study of individual longitudinal development instead of average group effects. Furthermore, LGM does not require complete data as it deals with missing data elegantly [38-40], and individual times between assessments can be included in the analysis.

Second, we used an active control condition that consisted of psycho-education. As psycho-education has been found to be effective for cancer-related fatigue [41], comparing AAF and eMBCT to PE is a strict way of evaluating these interventions. Interestingly, fatigue severity did not significantly reduce in the psycho-education condition. We speculate that this lack of effect may be due to the presentation of psycho-education, namely that it was the minimal control intervention. Participants were perhaps disappointed not being randomized to one of the guided interventions. However, mental health did significantly increase in psycho-education.

Third, as we wanted to study the intervention effect alone, we chose T0_b_ (after the eligibility check) as our baseline measurement instead of T0_a_ (at recruitment). As fatigue significantly reduced between T0_a_ and T0_b_(*n*=174, *t*=6.293, *df*=173, *P*<.001, *r*=.548), which was before any experimental intervention took place, choosing T0_b_ as baseline assessment prevented overestimation of the intervention effect.

Fourth, to make these results relevant for health care practice, we 1) chose not to exclude patients suffering from comorbidities that may also explain fatigue, 2) we included all cancer types and 3) included patients who were using hormone therapy or antidepressants during the study. We did not control for these contributing factors, except from the check that they were equally divided between the three conditions. In this way, the sample better represents the population for which these interventions were developed, and the results of effectiveness are better representative for health care practice. Although cancer type has not been found to be related to the persistence of fatigue [2], comorbidities (eg, thyroid dysfunction, cardiovascular diseases, rheumatism) and the use of hormone therapy or antidepressants are presumably influencing the level of fatigue [42,43]. Therefore, the effectiveness we found would probably be higher if we had chosen to study a population without comorbidities. In contrast, other researchers may choose exclusion criteria to limit confounding factors with the intervention effect to study the proof of concept. Although this decision is valid for research purposes, it consequently extends the gap between research findings and health care practice [44]. Therefore, we and others (eg, Treweek and Zwarenstein [45]) encourage researchers to study interventions that are intended to be applied in health care practice using a pragmatic randomized controlled trial (RCT) study design, with no strict exclusion criteria that extend the gap between research and health care practice.

In previous research it was found that female breast cancer patients with high education are well represented in the population that seeks support in mental health institutes specializing in psycho-oncology [46]. We therefore think the current sample, which has a large proportion of female breast cancer patients and a high level of education, is representative of this population, but less representative of the cancer population in general.

In line with the arguments above, clinicians and researchers should be cautious when comparing the effectivity results reported by different intervention studies (eg, for comparison Gielissen et al [7] and Abrahams et al [44]), because assessment points, normative groups, data analyses methods, and inclusion and exclusion criteria vary.

A limitation of this study was the unequal sample size of the conditions. As was previously reported in our trial design paper [14], the unequal sample size was partially caused by an error in the website’s randomization algorithm.

We noted several disadvantages of the RCT study design when evaluating these Web-based interventions. One limitation is that in an RCT design, the intervention is “frozen” in time, while technical applications evolve rapidly, resulting in the intervention being outdated when the effectiveness has been investigated. For example, the eMBCT webpage (developed in 2010) functioned poorly on a tablet, which led to treatment dropout of participants who used a tablet instead of a computer. Smaller and more elegant accelerometers have also come to the market, which affected the credibility of the devices that were used in this study. Another limitation of our study design is that we had to exclude participants based on scoring too low on CIS-FS at recruitment, despite the fact that they said they indeed suffered from extreme fatigue.

Another limitation was that the norm group that was used to calculate the percentage of clinically relevant improved participants was younger than our sample (norm group: mean age=45.9 years; SD=6.3 [35] versus our sample: mean age=55.1 years; SD=10.1) and only consisted of breast cancer patients. Ideally, we would have used a nonseverely fatigued group of cancer survivors, of approximately the same age as our sample, but this was not available in existing literature.

In conclusion, both the AAF and eMBCT are effective for managing fatigue severity compared to receiving psycho-educational emails. This is the first study that reported on the effectiveness of Web-based interventions for CCRF compared to an active control condition. The analytical methods of this study were new, and thereby added to the scientific knowledge on evaluating the clinical effectiveness of Web-based interventions. We are currently working on the analyses of a one-year follow-up [47]. To improve the interventions, we are also studying working mechanisms [48,49], and which baseline characteristics predict treatment outcomes. Additionally, to better attune interventions to the patients’ needs and reduce dropout, we performed qualitative analyses of semi-structured interviews with participants about their experiences with the interventions.

## Appendix

Multimedia Appendix 1 Recruitment over the course of time. The cumulative number of persons who applied to participate in the study and the number of included participants are shown over the course of recruitment time. The figure also shows when major recruitment actions were performed, such as when the improved Web page was launched.

Multimedia Appendix 2 Recruitment advertisement.

Multimedia Appendix 3 Informed consent.

Multimedia Appendix 4 Baseline characteristics of included participants (n=167).

Multimedia Appendix 5 Screenshots of the interventions.

Multimedia Appendix 6 Selection procedure and results of best model fit for fatigue severity (CIS-FS).

Multimedia Appendix 7 Calculations of proportion reliably changed participants.

## Abbreviations

AAF: Ambulant Activity Feedback
ANOVA: analysis of variance
CCRF: chronic cancer-related fatigue
CIS-FS: Checklist Individual Strength - Fatigue Severity subscale
CRF: cancer-related fatigue
DSM-IV-TR: Diagnostic and Statistical Manual of Mental Disorders, Fourth Edition, Text Revision
eMBCT: Web-based Mindfulness-Based Cognitive Therapy
FNK: Fitter na kanker
HADS: Hospital Anxiety and Depression Scale
LGM: Longitudinal Growth Modeling
NA: Negative Affect
PA: Positive Affect
RCI: reliable change index
RCT: randomized controlled trial
SD: standard deviation
