# Supplementary material for: Effectiveness of Two Web-Based Interventions for Chronic Cancer-Related Fatigue Compared to an Active Control Condition: Results of the “Fitter na kanker” Randomized Controlled Trial
Source: J Med Internet Res. 2017 Oct 19;19(10):e336. doi: 10.2196/jmir.7180 (PMC5668634; doi:10.2196/jmir.7180)
Supplement: Multimedia Appendix 1 [file jmir_v19i10e336_app1.pdf]

## Appendix 1. Recruitment over the course of time

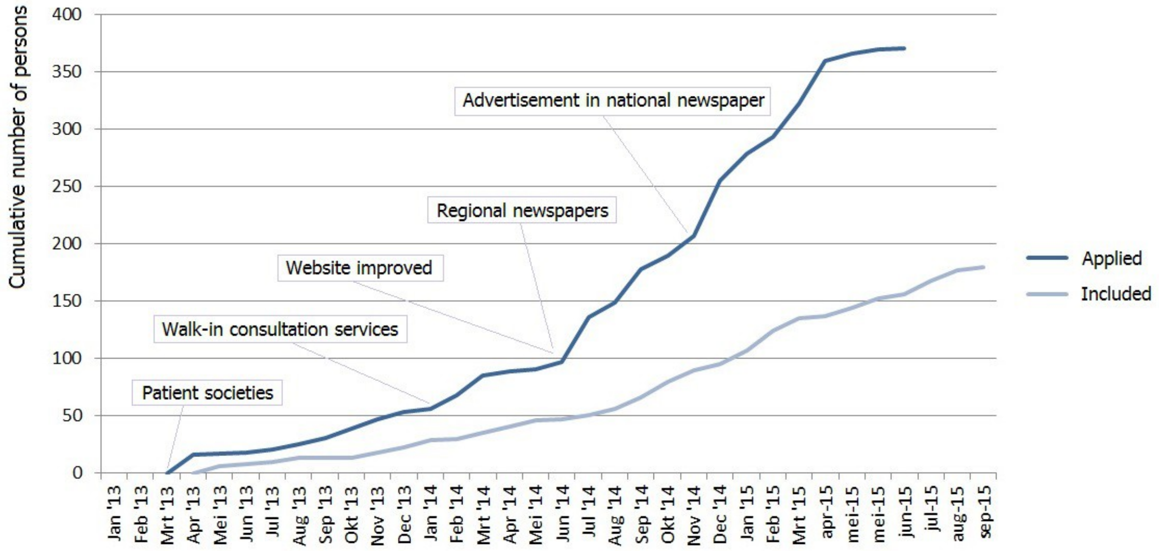


**Figure Appendix 1.** In this figure, the cumulative number of persons who applied to participate in the study and the number of included participants are shown over the course of recruitment time. It also shows when major recruitment actions were performed, such as when the improved Web page was launched.
