# Supplementary material for: Effectiveness of Two Web-Based Interventions for Chronic Cancer-Related Fatigue Compared to an Active Control Condition: Results of the “Fitter na kanker” Randomized Controlled Trial
Source: J Med Internet Res. 2017 Oct 19;19(10):e336. doi: 10.2196/jmir.7180 (PMC5668634; doi:10.2196/jmir.7180)
Supplement: Multimedia Appendix 2 [file jmir_v19i10e336_app2.pdf]

Appendix 2. Recruitment advertisement

Are you suffering from fatigue after cancer?

Have you had cancer in the past, but are you still suffering from fatigue? Then please apply for the study *Fitter na kanker*. In this study we will investigate two different internet interventions. Participants are offered an internet intervention that is guided by a psychologist or physiotherapist. For more information and application, go to www.fitternakanker.nl. *Fitter na kanker* is a collaboration between the Helen Dowling Institute and Roessingh Research and Development, and is subsidized by the Alpe d'HuZes/KWF-fund.
