# Supplementary material for: Effectiveness of Two Web-Based Interventions for Chronic Cancer-Related Fatigue Compared to an Active Control Condition: Results of the “Fitter na kanker” Randomized Controlled Trial
Source: J Med Internet Res. 2017 Oct 19;19(10):e336. doi: 10.2196/jmir.7180 (PMC5668634; doi:10.2196/jmir.7180)
Supplement: Multimedia Appendix 3 [file jmir_v19i10e336_app3.pdf]

Appendix 3. Informed consent

Informed consent for the study *Fitter na kanker* – Research on the effectiveness and working mechanisms of two different e-therapies for chronic fatigue after cancer

- I consent to participate in the above mentioned research.
- I have read the information letter for the study, and I have understood the purpose of the study and how much time-investment it will cost me.
- I have had plenty of time to think about my participation and have had the opportunity to ask questions. These questions have been answered satisfactorily.
- It is clear to me that I can withdraw from the study at any time, and that it has no consequences for me.
- I consent that the authorized persons of the Helen Dowling Institute and Roessingh Research and Development, members of the medical ethics committee, and competent authorities may have access to my research data. They are required to keep these research data secret.
- My research data are processed in scientific reports, but I understand that I cannot be recognized therein as a person. I know whom I can turn to with questions about the study.
- I know that if I were to decide to discontinue my participation in the study, my research data gathered prior to this decision may still be processed together with other data collected as part of this study.
- I give permission to the researchers of Fitter after cancer to contact me for any future research. I know I am free to participate in future research or not.
