# Supplementary material for: Effectiveness of Two Web-Based Interventions for Chronic Cancer-Related Fatigue Compared to an Active Control Condition: Results of the “Fitter na kanker” Randomized Controlled Trial
Source: J Med Internet Res. 2017 Oct 19;19(10):e336. doi: 10.2196/jmir.7180 (PMC5668634; doi:10.2196/jmir.7180)
Supplement: Multimedia Appendix 4 [file jmir_v19i10e336_app4.pdf]

Multimedia Appendix 1. Baseline characteristics of included participants (*n*=167)

	Mean (SD)/%		
Demographics	**AAF**	**eMBCT**	**PE**
Age (years)	56.45 (9.25)	51.36 (12.04)	56.54 (8.43)
Women	26,35 (n=44)	23,35 (n=39)	23,95 (n=40)
Dutch nationality	36,53 (n=61)	32,93 (n=55)	29,34 (n=49)
Living with partner and/or children	34,13 (n=57)	28,74 (n=48)	23,35 (n=39)
**Religious beliefs**			
No	22,16 (n=37)	22,75 (n=38)	19,16 (n=32)
Christian	10,18 (n=17)	7,78 (n=13)	8,38 (n=14)
Other	4,79 (n=8)	2,4 (n=4)	2,4 (n=4)
**Education**			
Low	1,2 (n=2)	2,4 (n=4)	0
Middle	13,17 (n=22)	11,38 (n=19)	7,19 (n=12)
High	22,75 (n=38)	19,16 (n=32)	22,75 (n=38)
**Employment**			
Paid job	17,96 (n=30)	11,98 (n=20)	12,57 (n=21)
Absent from work	7,78 (n=13)	4,19 (n=7)	6,59 (n=11)
Work ability	2.05 (1.40)	2.35 (2.08)	2.06 (1.49)
range	1-8	1-11	1-8
Medical history			
**Cancer diagnosis**			
Breast	14,97 (n=25)	14,97 (n=25)	17,37 (n=29)
Blood, bone marrow, Hodgkin's (except leukemia)	7,19 (n=12)	4,19 (n=7)	2,99 (n=5)
Reproductive organs	6,59 (n=11)	4,79 (n=8)	3,59 (n=6)
Digestive system	6,59 (n=11)	1,8 (n=3)	1,2 (n=2)
Head and neck	2,4 (n=4)	2,4 (n=4)	1,2 (n=2)
Urinary tract	1,8 (n=3)	1,2 (n=2)	2,4 (n=4)
Leukemia	0,6 (n=1)	2,99 (n=5)	1,2 (n=2)
Lung	2,4 (n=4)	0,6 (n=1)	0
Endocrine	1,2 (n=2)	0	1,2 (n=2)
Skin	1,2 (n=2)	0	1,2 (n=2)
CNS	0	1,2 (n=2)	0,6 (n=1)
Bone	1,8 (n=3)	1,2 (n=2)	0
**Time since first cancer diagnosis**			
<1 yr	0,6 (n=1)	0,6 (n=1)	1,2 (n=2)
1-2 yr	10,78 (n=18)	4,79 (n=8)	5,39 (n=9)
2-5 yr	8,38 (n=14)	13,77 (n=23)	13,17 (n=22)
> 5 yr	16,77 (n=28)	13,77 (n=23)	10,18 (n=17)
**Time since final cancer treatment**			
< 6 months	0 (n=0)	0,6 (n=1)	1,2 (n=2)
6 months - 1 year	4,79 (n=8)	4,79 (n=8)	4,19 (n=7)
1-2 year	9,58 (n=16)	7,78 (n=13)	7,19 (n=12)

2-5 year	8,38 (n=14)	10,78 (n=18)	14,37 (n=24)
> 5 year	13,17 (n=22)	8,98 (n=15)	2,99 (n=5)
**Cancer recurrence in the past**	0,6 (n=1)	2,99 (n=5)	4,19 (n=7)
**Hereditary form of cancer**	2,4 (n=4)	0 (n=0)	1,2 (n=2)
**Lymph nodes affected**	14,97 (n=25)	13,17 (n=22)	14,97 (n=25)
**Metastases**	4,79 (n=8)	4,19 (n=7)	4,19 (n=7)
**Good prognosis**	32,34 (n=54)	26,95 (n=45)	20,96 (n=35)
**Type of cancer treatment** [a]			
Surgery	31,74 (n=53)	22,16 (n=37)	25,15 (n=42)
Chemotherapy	24,55 (n=41)	22,75 (n=38)	22,75 (n=38)
Radiotherapy	17,37 (n=29)	4,19 (n=7)	19,16 (n=32)
Hormonal therapy	10,78 (n=18)	9,58 (n=16)	11,38 (n=19)
Immunotherapy	1,8 (n=3)	3,59 (n=6)	1,8 (n=3)
Stem cell or bone marrow transplantation	1,2 (n=2)	2,4 (n=4)	1,8 (n=3)
Other (oxygen, hyperthermia)	0	1,2 (n=2)	0,6 (n=1)
Operation only	5,99 (n=10)	0,6 (n=1)	2,99 (n=5)
Operation, radiotherapy and chemo, or more	10,18 (n=17)	10,18 (n=17)	13,77 (n=23)
**Comorbidity**			
No comorbidity	15,57 (n=26)	16,77 (n=28)	16,77 (n=28)
One comorbidity	13,17 (n=22)	11,98 (n=20)	8,38 (n=14)
More than one comorbidity	8,38 (n=14)	4,19 (n=7)	4,79 (n=8)
Lung	13,89 (n=5)	22,22 (n=6)	0 (n=0)
Cardiovascular	11,11 (n=4)	3,7 (n=1)	15 (n=3)
Rheumatism	33,33 (n=12)	7,41 (n=2)	22,73 (n=5)
Physical injury	25 (n=9)	3,7 (n=1)	22,73 (n=5)
Neurological	16,67 (n=6)	7,41 (n=2)	36,36 (n=8)
Organs (kidney, liver)	22,22 (n=8)	14,81 (n=4)	9,09 (n=2)
Thyroid	8,33 (n=3)	25,93 (n=7)	13,64 (n=3)
Other	13,89 (n=5)	33,33 (n=9)	22,73 (n=5)
**BMI**			
<25	11,38 (n=19)	14,37 (n=24)	13,77 (n=23)
25-30	13,17 (n=22)	10,78 (n=18)	8,98 (n=15)
>30	6,59 (n=11)	5,99 (n=10)	4,79 (n=8)
**Medication use between $T0_b$ and T2**			
Hormonal therapy	4,79 (n=8)	8,98 (n=15)	7,78 (n=13)
Changed hormone therapy use	0,6 (n=1)	1,8 (n=3)	0,6 (n=1)
Antidepressants	1,8 (n=3)	0,6 (n=1)	1,8 (n=3)
Changed antidepressants use	0 (n=0)	0,6 (n=1)	0 (n=0)
**Duration of fatigue**			
0- 1 year	7,19 (n=12)	5,99 (n=10)	8,38 (n=14)
1-5 years	14,97 (n=25)	14,37 (n=24)	16,77 (n=28)

> 5 years	14,37 (n=24)	12,57 (n=21)	4,79 (n=8)
Help received			
Psychological counseling in the past	19,16 (n=32)	14,97 (n=25)	16,17 (n=27)
Has received help to cope with cancer in the past	24,55 (n=41)	22,75 (n=38)	21,56 (n=36)
No experience with attention-focused exercises, such as meditation or yoga	14,37 (n=24)	17,37 (n=29)	14,37 (n=24)
Followed any other form of psychological care for fatigue at baseline	5,39 (n=9)	1,2 (n=2)	1,2 (n=2)
Fatigue			
CIS-FS $T0_a$	44.94 (5.63)	45.67 (5.04)	45.44 (5.46)
range	35-56	25-55	35-56
CIS-FS $T0_b$	43.24 (6.76)	42.84 (7.95)	40.34 (9.00)
range	24-55	22-55	19-56
Mental Health			
PANAS total	52.15 (6.26)	49.89 (6.27)	50.14 (7.98)
range	42-71	38-64	35-68
Negative affect	20.94 (7.68)	21.21 (6.86)	20.74 (8.80)
range	10-43	10-34	10-43
Positive affect	31.21 (7.32)	28.69 (6.88)	29.40 (7.71)
range	12-50	15-43	13-48
HADS total	13.77(7.32)	14.35 (5.73)	14.74 (7.41)
range	0-30	4-27	1-33
≥20 at baseline	15	9	12
Compliance Average time (weeks) between $T0_b$ and M6	11.42 (2.55)	12.56 (3.23)	9.21 (1.47)
range	7-18	8-23	7-13

[a] Numbers do not sum to n=167, as some participants had undergone multiple cancer treatments. Abbreviations: CIS-FS=Checklist Individual Strength-fatigue severity subscale; HADS=Hospital Anxiety and Depression Scale; PANAS=Positive and Negative Affect Schedule.
