# Supplementary material for: Effectiveness of Two Web-Based Interventions for Chronic Cancer-Related Fatigue Compared to an Active Control Condition: Results of the “Fitter na kanker” Randomized Controlled Trial
Source: J Med Internet Res. 2017 Oct 19;19(10):e336. doi: 10.2196/jmir.7180 (PMC5668634; doi:10.2196/jmir.7180)
Supplement: Multimedia Appendix 5 [file jmir_v19i10e336_app5.pdf]

Appendix 5. Screenshots of the interventions

Additional screenshot of the Web portal for ambulant activity feedback therapy (Dutch), as well as an image of the accelerometer, used to monitor physical activity bahavior.

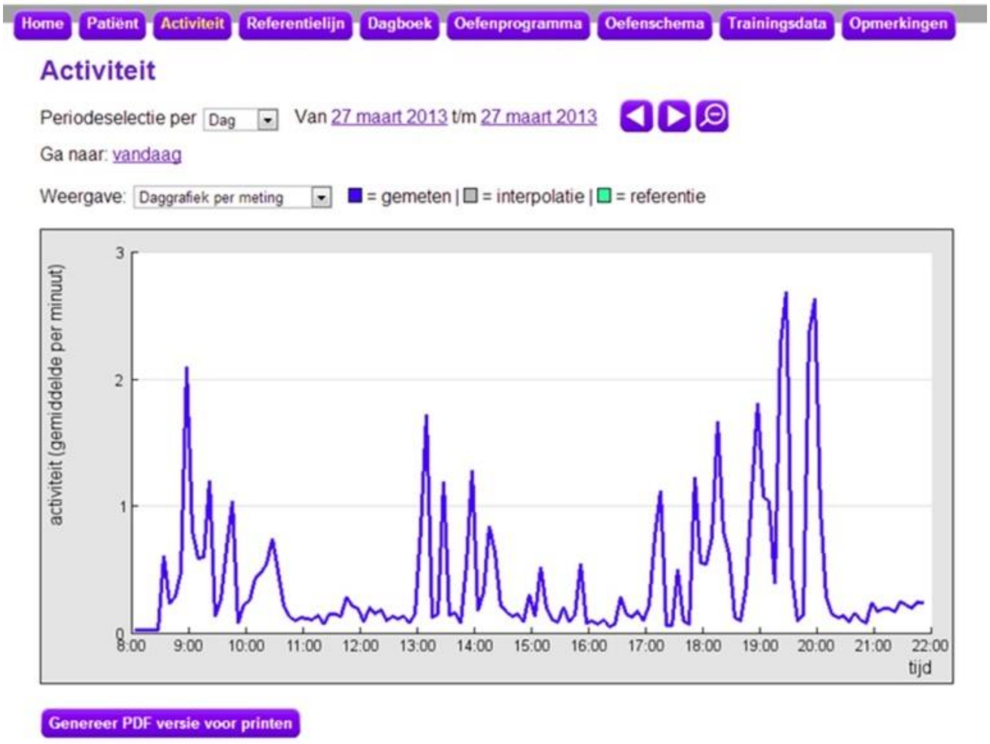

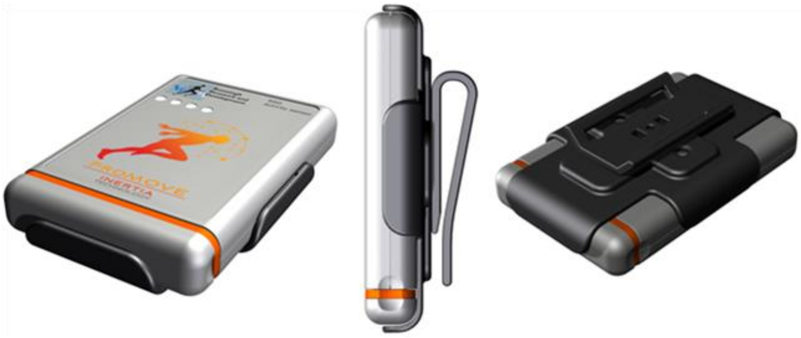

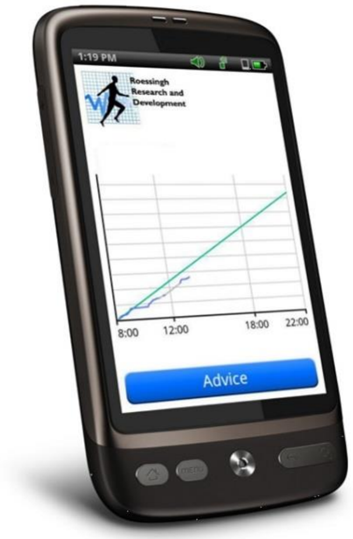

Additional screenshot of the patient Web portal for eMBCT (Dutch).

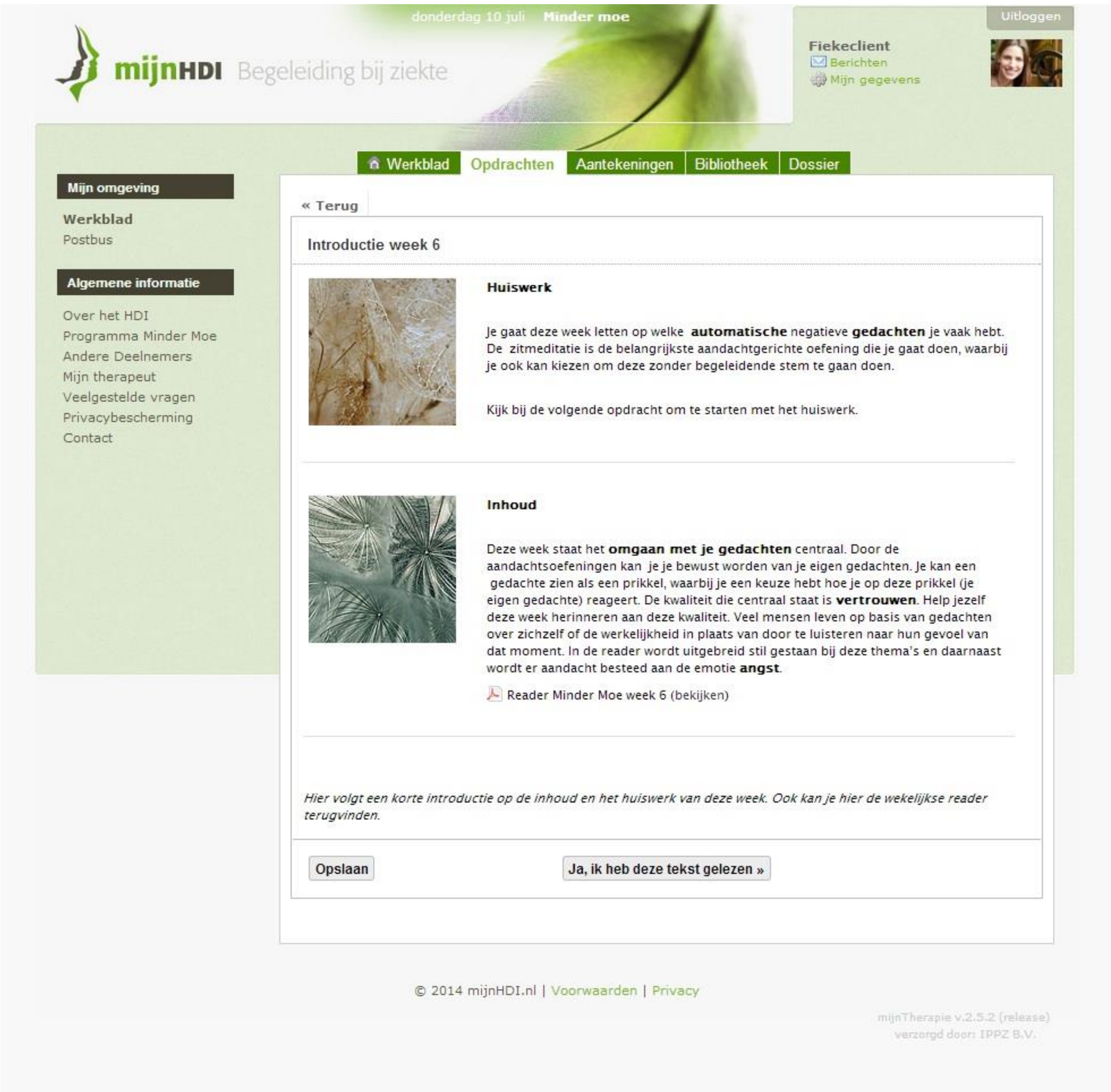

donderdag 10 juli **Minder moe**

mijnHDI Begeleiding bij ziekte

Uitloggen

Fiekeclient
Berichten
Mijn gegevens

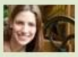

Werkblad | Opdrachten | Aantekeningen | Bibliotheek | Dossier

Mijn omgeving

Werkblad
Postbus

Algemene informatie

Over het HDI
Programma Minder Moe
Andere Deelnemers
Mijn therapeut
Veelgestelde vragen
Privacybescherming
Contact

« Terug

Aandacht voor de ademhaling (dagelijks)

Dagboek

Voorbeeld - Dagboek van Willem

Dag 1

Oefening – Aandacht voor de ademhaling

Ervaringen/ opmerkingen:

Leuk om te merken dat het steeds weer lukt om de aandacht te herleiden. Onwillekeurig dwalen de gedachten weg, soms zo geleidelijk dat je het nauwelijks merkt.

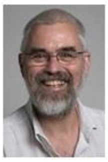

Mijn dagboek

Speel één van de twee geluidsbestanden 'Aandacht voor de ademhaling' af, en noteer na het oefenen wat je ervaren hebt.

Aandacht voor de ademhaling (14 min. - vrouw)

▶ 00:00 00:00

Download dit bestand

Aandacht voor de ademhaling (13 min. - stem man)

▶ 00:00 00:00

Download dit bestand

Dag 1 - Aandacht voor de ademhaling

Mijn ervaring / opmerkingen:

Dag 2 - Aandacht voor de ademhaling

Mijn ervaring / opmerkingen:

Example of an information letter for the minimal intervention control condition (Dutch).

Fitter na kanker

Informatie week 1

Vermoeidheid bij en na kanker

Vermoeidheid bij of na kanker komt veel voor. Bijna iedereen heeft er tijdens de behandeling van kanker last van. Ongeveer één op de drie patiënten blijft ook na de behandeling langdurig erg moe. Dus ook als er geen lichamelijke oorzaken voor vermoeidheid meer aan te wijzen zijn, kan iemand dus moe blijven. Daar zijn verschillende oorzaken voor. Met behulp van begeleiding is het mogelijk om deze oorzaken te ontdekken en te leren er beter mee om te gaan. Hierdoor kan herstel mogelijk worden.

Waarom blijf ik zo moe?

In de volgende e-mails zullen er enkele verklaringen genoemd worden voor chronische vermoeidheid. Een aantal hiervan komen in de e-mails van komende weken uitgebreider aan bod.

Lichamelijke gevolgen van de behandeling

Tijdens de ziekte en de behandelingen krijgt een lichaam het zwaar te verduren. Het gevecht tegen kanker en het herstellen van de behandeling kost energie. Ook kan de behandeling nawerkingen hebben waardoor u nog lange tijd moe blijft. Wij raden aan om uw vermoeidheidsklachten altijd eerst met de (huis)arts te bespreken, zodat een lichamelijke behandelbare oorzaak uitgesloten kan worden.

Angst

Door de kanker kun je angstiger geworden zijn. Angst kan heel moe maken. Wij hebben allemaal onze manieren om met angst om te gaan. Een van de meest voorkomende manieren is dat we hem niet willen voelen en proberen er zo min mogelijk aan te denken. Vaak helpt dat, maar soms kost het wegduwen van angst zoveel energie dat je daar erg moe van wordt. Het helpt om te ontdekken wat jouw manier is om met angst om te gaan. Je kunt vervolgens leren om er anders mee om te gaan, zodat je minder last hebt van vermoeidheid.
