# Supplementary material for: Effectiveness of Two Web-Based Interventions for Chronic Cancer-Related Fatigue Compared to an Active Control Condition: Results of the “Fitter na kanker” Randomized Controlled Trial
Source: J Med Internet Res. 2017 Oct 19;19(10):e336. doi: 10.2196/jmir.7180 (PMC5668634; doi:10.2196/jmir.7180)
Supplement: Multimedia Appendix 6 [file jmir_v19i10e336_app6.pdf]

Appendix 6. Selection procedure and results of best model fit for fatigue severity (CIS-FS)

The figure below shows the various tested models for fatigue severity trajectory (CIS-FS). We tested if a linear or linear and quadratic slope would best fit the data. As the timing of assessments varied between participants (see lower part of the figure for the variation per assessment), we also ran a model with individually varying times of assessments, known as timescores [51] instead of the fixed factor loadings reflecting our study design [0,1,2,3,4,8]. In that way, we were able to include in the model the exact time points when a participant completed the assessment. Also, for the CIS-FS data, we tested whether a piece-wise model would better fit the data than a non-piece wise model, thus a separate slope during the intervention (slope 1: $T0_b$–M3–M6–M9) and a separate slope after the intervention (slope 2: M9–T1–T2) in favor of one slope ($T0_b$–M3–M6–M9–T1–T2). The model selection procedures for HADS and PANAS were similar, except that no piece-wise model was estimated because the secondary outcome measures were assessed three times ($T0_b$–T1–T2). Therefore, only the procedure for CIS-FS is shown in this appendix.

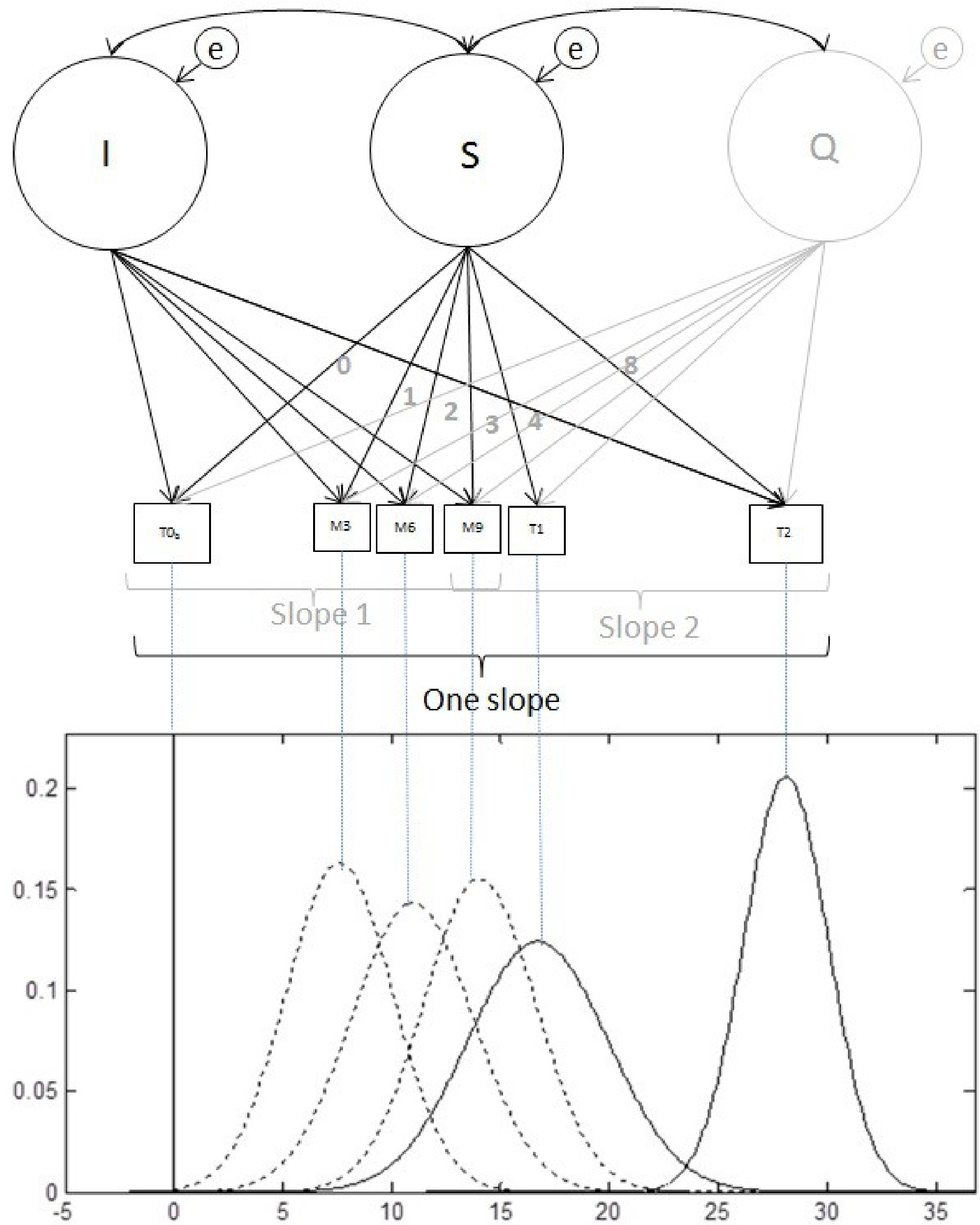

Figure Appendix 6. Illustration of the piece-wise latent growth curve model of CIS-FS with timescores. We compared models with: (1) linear or quadratic growth terms; (2) with or without individual time between assessments (timescores), and (3) one slope or two piece-wise model (slope 1 and 2). The factor loadings in this figure make up the metric of time reflecting our study design [0,1,2,3,4,8]. The lower part of the figure shows the estimated density plots of the timing

(weeks) of assessments (timescores) on the x-axis, and probability on the y-axis. Abbreviations: I=intercept, S=linear slope, Q=quadratic slope, e= residual variance.

The average time between assessments in weeks are shown below.

Timeframe	Mean	Standard deviation	range
T0b-M3	7.60	2.443	3-18
T0b-M6	10.96	2.785	7-23
T0b-M9	14.04	2.567	10-24
T0b-T1	16.71	3.215	12-29
T0b-T2	28.10	1.936	23-37

In the table below, the AIC and BIC model results are shown for each model tested. These models differed in terms of:

- slope: linear (is) or linear and quadratic (isq)
- model: piece-wise (PW) or one trend line (*LGM*)
- time modeling: with or without timescores (*TSC*).

In the second step, model fit was improved by imposing constraints (see abbreviations below). The model with the lowest fit indices (AIC and BIC sumscore), and secondly the most parsimonious model, resulting in model selection of *B2.LGM_CIS_isq_TSC.inp* for the effectiveness analysis. When the variances or residual variances of the means and slopes were not significantly different from zero in all three conditions, these were constrained to be equal between conditions to see if this would improve model fit. When error messages for the residual variances were present, these were constrained to be equal between conditions.

STEP 1, run all models without constraints

name input Mplus	***constraint***			***AIC+BIC***	***warnings/errors***
A1.LGM_CIS_is.inp	none	(AIC)	5.698.798	11.500.490	none
		(BIC)	5.801.692		
B1.LGM_CIS_isq.inp	none	(AIC)	5.597.179	11.334.667	WARNING: THE RESIDUAL COVARIANCE MATRIX (THETA) IN GROUP eMBCT AND PE IS NOT POSITIVE DEFINITE. THIS COULD INDICATE A NEGATIVE VARIANCE/RESIDUAL VARIANCE FOR AN OBSERVED VARIABLE, A CORRELATION GREATER OR EQUAL TO ONE BETWEEN TWO OBSERVED VARIABLES, OR A LINEAR DEPENDENCY AMONG MORE THAN TWO OBSERVED VARIABLES.CHECK THE RESULTS SECTION FOR MORE INFORMATION. PROBLEM INVOLVING VARIABLE T2CISZ. WARNING: THE LATENT VARIABLE COVARIANCE MATRIX (PSI) IN GROUP EMBCT IS NOT POSITIVE DEFINITE. THIS COULD INDICATE A NEGATIVE VARIANCE/RESIDUAL VARIANCE FOR A LATENT VARIABLE, A CORRELATION GREATER OR EQUAL TO ONE BETWEEN TWO LATENT VARIABLES, OR A LINEAR DEPENDENCY AMONG MORE THAN TWO LATENT VARIABLES. CHECK THE TECH4 OUTPUT FOR MORE INFORMATION. PROBLEM INVOLVING VARIABLE S.
		(BIC)	5.737.488		
A2.LGM_CIS_is_TSC.inp	none	(AIC)	5.667.421	11.437.736	none
		(BIC)	5.770.315		
B2.LGM_CIS_isq_TSC.inp	none	(AIC)	error	0	THE MODEL ESTIMATION DID NOT TERMINATE NORMALLY DUE TO AN ILL-CONDITIONED FISHER

		(BIC)	error		INFORMATION MATRIX. CHANGE YOUR MODEL AND/OR STARTING VALUES. THE MODEL ESTIMATION DID NOT TERMINATE NORMALLY DUE TO A NON-POSITIVE DEFINITE FISHER INFORMATION MATRIX. THIS MAY BE DUE TO THE STARTING VALUES BUT MAY ALSO BE AN INDICATION OF MODEL NONIDENTIFICATION. THE CONDITION NUMBER IS 0.479D-10. THE STANDARD ERRORS OF THE MODEL PARAMETER ESTIMATES COULD NOT BE COMPUTED. THIS IS OFTEN DUE TO THE STARTING VALUES BUT MAY ALSO BE AN INDICATION OF MODEL NONIDENTIFICATION. CHANGE YOUR MODEL AND/OR STARTING VALUES. PROBLEM INVOLVING THE FOLLOWING PARAMETER: Parameter 36, Group PE: T2CISZ

name input Mplus	***constraint***			***AIC+BIC***	***warnings/errors***
C1.PW_CIS_is_is.inp	none	(AIC)	5.600.617	11.341.544	WARNING: THE LATENT VARIABLE COVARIANCE MATRIX (PSI) IN GROUP EMBCT IS NOT POSITIVE DEFINITE. THIS COULD INDICATE A NEGATIVE VARIANCE/ RESIDUAL VARIANCE FOR A LATENT VARIABLE, A CORRELATION GREATER OR EQUAL TO ONE BETWEEN TWO LATENT VARIABLES, OR A LINEAR DEPENDENCY AMONG MORE THAN TWO LATENT VARIABLES. CHECK THE TECH4 OUTPUT FOR MORE INFORMATION. PROBLEM INVOLVING VARIABLE S1. WARNING: THE RESIDUAL COVARIANCE MATRIX (THETA) IN GROUP PE IS NOT POSITIVE DEFINITE. THIS COULD INDICATE A NEGATIVE VARIANCE/RESIDUAL VARIANCE FOR AN OBSERVED VARIABLE, A CORRELATION GREATER OR EQUAL TO ONE BETWEEN TWO OBSERVED VARIABLES, OR A LINEAR DEPENDENCY AMONG MORE THAN TWO OBSERVED VARIABLES. CHECK THE RESULTS SECTION FOR MORE INFORMATION. PROBLEM INVOLVING VARIABLE T2CISZ.
		(BIC)	5.740.927		
D1.PW_CIS_isq_is.inp	none	(AIC)	5.579.688	11.346.455	WARNING: THE RESIDUAL COVARIANCE MATRIX (THETA) IN GROUP AAF IS NOT POSITIVE

		(BIC)	5.766.767		DEFINITE. THIS COULD INDICATE A NEGATIVE VARIANCE/RESIDUAL VARIANCE FOR AN OBSERVED VARIABLE, A CORRELATION GREATER OR EQUAL TO ONE BETWEEN TWO OBSERVED VARIABLES, OR A LINEAR DEPENDENCY AMONG MORE THAN TWO OBSERVED VARIABLES. CHECK THE RESULTS SECTION FOR MORE INFORMATION. PROBLEM INVOLVING VARIABLE TBCISZ. WARNING: THE LATENT VARIABLE COVARIANCE MATRIX (PSI) IN GROUP EMBCT IS NOT POSITIVE DEFINITE. THIS COULD INDICATE A NEGATIVE VARIANCE/ RESIDUAL VARIANCE FOR A LATENT VARIABLE, A CORRELATION GREATER OR EQUAL TO ONE BETWEEN TWO LATENT VARIABLES, OR A LINEAR DEPENDENCY AMONG MORE THAN TWO LATENT VARIABLES. CHECK THE TECH4 OUTPUT FOR MORE INFORMATION. PROBLEM INVOLVING VARIABLE I. WARNING: THE RESIDUAL COVARIANCE MATRIX (THETA) IN GROUP PE IS NOT POSITIVE DEFINITE. THIS COULD INDICATE A NEGATIVE VARIANCE/RESIDUAL VARIANCE FOR AN OBSERVED VARIABLE, A CORRELATION GREATER OR EQUAL TO ONE BETWEEN TWO OBSERVED VARIABLES, OR A LINEAR DEPENDENCY AMONG MORE THAN TWO OBSERVED VARIABLES. CHECK THE RESULTS SECTION FOR MORE INFORMATION. PROBLEM INVOLVING VARIABLE TBCISZ.
C2.PW_CIS_is_is_TSC.inp	none	(AIC)<br>(BIC)	5.670.371<br>5.810.681	11.481.052	none
D2.PW_CIS_isq_is_TSC.inp	none	(AIC)<br>(BIC)	5.684.750<br>5.871.830	11.556.580	WARNING: THE MODEL ESTIMATION HAS REACHED A SADDLE POINT OR A POINT WHERE THE OBSERVED AND THE EXPECTED INFORMATION MATRICES DO NOT MATCH. AN ADJUSTMENT TO THE ESTIMATION OF THE INFORMATION MATRIX HAS BEEN MADE. THE CONDITION NUMBER IS -0.325D-02. THE PROBLEM MAY ALSO BE RESOLVED BY DECREASING THE VALUE OF THE MCONVERGENCE OR LOGCRITERION OPTIONS OR BY CHANGING THE STARTING VALUES OR BY USING THE MLF ESTIMATOR.

STEP 2, improve fit with adding constraints based on output in step1:

- When variances were non-significant in all groups they were constraint to 0.
- When residual variances were non-significant, they were constraint to be equal between groups.

- When residual variances were non-significant, they were constraint to be equal between groups

name input Mplus	***constraint***			***AIC+BIC***	***warnings/errors***
A1.LGM_CIS_is.inp	s@0	(AIC)<br>(BIC)	5.697.432<br>5.781.617	11.479.049	none
A2.LGM_CIS_is_TSC.inp	s@0	(AIC)<br>(BIC)	5.672.659<br>5.756.844	11.429.503	none
B1.LGM_CIS_isq.inp	res var (1)	(AIC)<br>(BIC)	5.586.788<br>5.674.092	11.260.880	none
B2.LGM_CIS_isq_TSC.inp	q@0	(AIC)<br>(BIC)	5.657.207<br>5.769.455	11.426.662	WARNING: THE MODEL ESTIMATION HAS REACHED A SADDLE POINT OR A POINT WHERE THE OBSERVED AND THE EXPECTED INFORMATION MATRICES DO NOT MATCH. AN ADJUSTMENT TO THE ESTIMATION OF THE INFORMATION MATRIX HAS BEEN MADE. THE CONDITION NUMBER IS -0.102D-06. THE PROBLEM MAY ALSO BE RESOLVED BY DECREASING THE VALUE OF THE MCONVERGENCE OR LOGCRITERION OPTIONS OR BY CHANGING THE STARTING VALUES OR BY USING THE MLF ESTIMATOR.
**B2.LGM_CIS_isq_TSC.inp**	**s@0**	(AIC)<br>(BIC)	error<br>error	0	THE STANDARD ERRORS OF THE MODEL PARAMETER ESTIMATES COULD NOT BE COMPUTED. THIS IS OFTEN DUE TO THE STARTING VALUES BUT MAY ALSO BE AN INDICATION OF MODEL NONIDENTIFICATION. CHANGE YOUR MODEL AND/OR STARTING VALUES.
B2.LGM_CIS_isq_TSC.inp	res var (1)	(AIC)<br>(BIC)	5.581.389<br>5.668.693	11.250.082	none
B2.LGM_CIS_isq_TSC.inp	q@0 en s@0	(AIC)	5.668.360	11.430.260	none

		(BIC)	5.761.900		
C1.PW_CIS_is_is.inp	[s2](1)	(AIC)	5.597.636	11.329.346	WARNING: THE LATENT VARIABLE COVARIANCE MATRIX (PSI) IN GROUP EMBCT IS NOT POSITIVE DEFINITE. THIS COULD INDICATE A NEGATIVE VARIANCE/ RESIDUAL VARIANCE FOR A LATENT VARIABLE, A CORRELATION GREATER OR EQUAL TO ONE BETWEEN TWO LATENT VARIABLES, OR A LINEAR DEPENDENCY AMONG MORE THAN TWO LATENT VARIABLES. CHECK THE TECH4 OUTPUT FOR MORE INFORMATION. PROBLEM INVOLVING VARIABLE S1. WARNING: THE RESIDUAL COVARIANCE MATRIX (THETA) IN GROUP PE IS NOT POSITIVE DEFINITE. THIS COULD INDICATE A NEGATIVE VARIANCE/RESIDUAL VARIANCE FOR AN OBSERVED VARIABLE, A CORRELATION GREATER OR EQUAL TO ONE BETWEEN TWO OBSERVED VARIABLES, OR A LINEAR DEPENDENCY AMONG MORE THAN TWO OBSERVED VARIABLES. CHECK THE RESULTS SECTION FOR MORE INFORMATION. PROBLEM INVOLVING VARIABLE T2CISZ.
		(BIC)	5.731.710		
C1.PW_CIS_is_is.inp	[s2](1) en res var (1)	(AIC)	6.266.864	12.611.678	none
		(BIC)	6.344.814		
C1.PW_CIS_is_is.inp	res var (1)	(AIC)	5.589.511	11.266.325	none
		(BIC)	5.676.814		
C2.PW_CIS_is_is_TSC.inp	s2@0	(AIC)	5.675.016	11.462.280	none
		(BIC)	5.787.264		
C2.PW_CIS_is_is_TSC.inp	s1@0 en s2@0	(AIC)	5.666.057	11.425.654	none
		(BIC)	5.759.597		

D1.PW_CIS_isq_is.inp	[s2](1)	(AIC)	5.576.497	11.333.838	WARNING: THE RESIDUAL COVARIANCE MATRIX (THETA) IN GROUP AAF IS NOT POSITIVE DEFINITE. THIS COULD INDICATE A NEGATIVE VARIANCE/RESIDUAL VARIANCE FOR AN OBSERVED VARIABLE, A CORRELATION GREATER OR EQUAL TO ONE BETWEEN TWO OBSERVED VARIABLES, OR A LINEAR DEPENDENCY AMONG MORE THAN TWO OBSERVED VARIABLES. CHECK THE RESULTS SECTION FOR MORE INFORMATION. PROBLEM INVOLVING VARIABLE TBCISZ. WARNING: THE LATENT VARIABLE COVARIANCE MATRIX (PSI) IN GROUP EMBCT IS NOT POSITIVE DEFINITE. THIS COULD INDICATE A NEGATIVE VARIANCE/ RESIDUAL VARIANCE FOR A LATENT VARIABLE, A CORRELATION GREATER OR EQUAL TO ONE BETWEEN TWO LATENT VARIABLES, OR A LINEAR DEPENDENCY AMONG MORE THAN TWO LATENT VARIABLES. CHECK THE TECH4 OUTPUT FOR MORE INFORMATION. PROBLEM INVOLVING VARIABLE I. WARNING: THE RESIDUAL COVARIANCE MATRIX (THETA) IN GROUP PE IS NOT POSITIVE DEFINITE. THIS COULD INDICATE A NEGATIVE VARIANCE/RESIDUAL VARIANCE FOR AN OBSERVED VARIABLE, A CORRELATION GREATER OR EQUAL TO ONE BETWEEN TWO OBSERVED VARIABLES, OR A LINEAR DEPENDENCY AMONG MORE THAN TWO OBSERVED VARIABLES. CHECK THE RESULTS SECTION FOR MORE INFORMATION. PROBLEM INVOLVING VARIABLE TBCISZ.
		(BIC)	5.757.341		
D1.PW_CIS_isq_is.inp	res var (1) en [s2](1)	(AIC)	6.155.517	12.435.753	none
		(BIC)	6.280.236		
D1.PW_CIS_isq_is.inp	res var (1) en q1@0	(AIC)	5.564.601	11.225.860	none
		(BIC)	5.661.259		
D1.PW_CIS_isq_is.inp	res var (1)	(AIC)	5.573.097	11.280.268	none
		(BIC)	5.707.171		
D2.PW_CIS_isq_is_TSC.inp	s1@0	(AIC)	error	0	THE STANDARD ERRORS OF THE MODEL PARAMETER ESTIMATES COULD NOT BE COMPUTED. THIS IS OFTEN DUE TO THE

		(BIC)	error		STARTING VALUES BUT MAY ALSO BE AN INDICATION OF MODEL NONIDENTIFICATION. CHANGE YOUR MODEL AND/OR STARTING VALUES.
D2.PW_CIS_isq_is_TSC.inp	s2@0	(AIC)	5.685.188	11.520.040	WARNING: THE MODEL ESTIMATION HAS REACHED A SADDLE POINT OR A POINT WHERE THE OBSERVED AND THE EXPECTED INFORMATION MATRICES DO NOT MATCH. AN ADJUSTMENT TO THE ESTIMATION OF THE INFORMATION MATRIX HAS BEEN MADE. THE CONDITION NUMBER IS -0.197D-01. THE PROBLEM MAY ALSO BE RESOLVED BY DECREASING THE VALUE OF THE MCONVERGENCE OR LOGCRITERION OPTIONS OR BY CHANGING THE STARTING VALUES OR BY USING THE MLF ESTIMATOR.
		(BIC)	5.834.852		
D2.PW_CIS_isq_is_TSC.inp	s1@0 en s2@0	(AIC)	error	0	THE ESTIMATED COVARIANCE MATRIX FOR AAF IS NOT POSITIVE DEFINITE AS IT SHOULD BE. COMPUTATION COULD NOT BE COMPLETED. PROBLEM INVOLVING VARIABLE Q1. THE CORRELATION BETWEEN Q1 AND I IS -1.071 THE RESIDUAL CORRELATION BETWEEN Q1 AND I IS -1.071 THE PROBLEM MAY BE RESOLVED BY SETTING ALGORITHM=EM AND MCONVERGENCE TO A LARGE VALUE. THE MODEL ESTIMATION DID NOT TERMINATE NORMALLY DUE TO AN ERROR IN THE COMPUTATION. CHANGE YOUR MODEL AND/OR STARTING VALUES
		(BIC)	error		

<u>Abbreviations</u>

LGM	latent growth model, thus one trend line
PW	Piece-wise latent growth model
is	slope is linear
isq	slope is both linear and quadratic
TSC	with individual timescores
no TSC	model-based timescores [0,1,2,3,4,8]
res var (1)	residual variances constrained to be equal between groups
s@0	linear slope variance is constrained to 0 in all groups
q@0	quadratic slope variance is constrained to 0 between groups
[s](1)	slope mean is constrained to be equal between groups

BIC	Bayesian Information Criterion
AIC	Akaike Information Criterion
