# Supplementary material for: Effectiveness of Two Web-Based Interventions for Chronic Cancer-Related Fatigue Compared to an Active Control Condition: Results of the “Fitter na kanker” Randomized Controlled Trial
Source: J Med Internet Res. 2017 Oct 19;19(10):e336. doi: 10.2196/jmir.7180 (PMC5668634; doi:10.2196/jmir.7180)
Supplement: Multimedia Appendix 7 [file jmir_v19i10e336_app7.pdf]

Appendix 7. Calculations of proportion reliably changed participants

The proportion of participants who were clinically relevantly changed on fatigue severity (CIS-FS) was calculated using the reliable change index (RCI) [34,35]. The Reliable change index (RCI) was calculated according to the method proposed by Jacobson & Truax [34] (see also Maassen [35]).

The RCI per individual was calculated by applying the following formula:

(3) $$RCI = \frac{X_2 - X_1}{S_{diff}} RCI = \frac{X_2 - X_1}{S_{diff}}$$

where $X_2 - X_1$ is the individual change score of CIS-FS between $T0_b$ and T2. To be able to calculate the RCI for all participants – so also for participants who had missing data at T2 – we used the estimated intercepts of CIS-FS at T2 of the best fitting model (see Appendix 6 for model selection). These intercepts were obtained using the FSCORE function in the SAVE command of Mplus.

S_{diff} was calculated by applying the following formula:

(4) $$S_{diff} = \sqrt{2(SE)^2}\, S_{diff} = \sqrt{2(SE)^2}$$

where *SE* is denoted by:

(5) $$SE = SD_1 \sqrt{1 - r_{xx}}\, SE = SD_1 \sqrt{1 - r_{xx}}$$

where SD_1 is the standard deviation of the norm group and r_{xx} is the test-retest reliability of the CIS-fatigue severity subscale.

The norm group consisted of non-fatigued cancer survivor (n=93) [36] (Mean CIS-FS = 19.6, *SD* = 8.4). The cut-off norm group was *M* +1 *SD* = 28.0. The test-retest reliability was r_{xx} = 0.88 based on Vercoulen et al [52].

Then, the proportion of clinically relevant improved participants was calculated based on the following definitions:

Improved: passed RCI in direction of fatigue reduction, thus RCI <- 1.96

Unchanged: did not pass the RCI, thus - 1.96 < RCI < 1.96.

Deteriorated: passed RCI in direction of fatigue increase, thus RCI >1.96

Recovered: passed RCI in direction of fatigue reduction, thus RCI <- 1.96 and CIS-FS T2 was below the cut-off point of the norm group, so below 28.
